# Supplementary material for: Complexome profiling of the Chlamydomonas psb28 mutant reveals TEF5 as an early PSII assembly factor
Source: Plant Cell. 2025 Mar 17;37(6):koaf055. doi: 10.1093/plcell/koaf055 (PMC12164590; doi:10.1093/plcell/koaf055)
Supplement: koaf055_Supplementary_Data [file koaf055_supplementary_data.zip › psb28_interactive.zip!/plots/intensity/qualityPlots/peptides/MCCA_Cre06.g278098.t1.1.html]

### Cre06.g278098.t1.1

Trivial name: MCCA  
SeqCoverage [median]: 3.80  
Mass: 82.19 kDa  
  
 

| Sequence | MissedCleavage | Mass [Da] | wtIntensity | muIntensity | Score |
| --- | --- | --- | --- | --- | --- |
| IIEEAPAPGLPEEFHR | 0 | 1803.92 | 0.00 | 1126.34 | 37.55 |
| TPAAAVTFANAPVR | 0 | 1384.75 | 379.79 | 0.00 | 83.00 |
|  |  |  | 379.8 | 1126.3 |  |
